# Link between cognitive polygenic risk scores and clinical progression after a first-psychotic episode

**DOI:** 10.1017/S0033291722001544

**Published:** 2023-07

**Authors:** Alex G. Segura, Gisela Mezquida, Albert Martínez-Pinteño, Patricia Gassó, Natalia Rodriguez, Lucía Moreno-Izco, Silvia Amoretti, Miquel Bioque, Antonio Lobo, Ana González-Pinto, Alicia García-Alcon, Alexandra Roldán-Bejarano, Eduard Vieta, Elena de la Serna, Alba Toll, Manuel J. Cuesta, Sergi Mas, Miquel Bernardo, PEPs Group

**Affiliations:** 1 Department of Clinical Foundations, Pharmacology Unit, University of Barcelona, Barcelona, Spain; 2 Barcelona Clínic Schizophrenia Unit, Neuroscience Institute Hospital Clínic de Barcelona, Barcelona, Spain; 3 Centro de Investigación Biomédica en Red de Salud Mental (CIBERSAM), Madrid, Spain; 4 Institut d'investigacions Biomèdiques August Pi i Sunyer (IDIBAPs), Barcelona, Spain; 5 Department of Psychiatry, Complejo Hospitalario de Navarra, Pamplona, Spain; 6 Navarra Institute for Health Research (IdiSNA), Pamplona, Spain; 7 Group of Psychiatry, Mental Health and Addictions, Psychiatric Genetics Unit, Vall d'Hebron Research Institute (VHIR), Barcelona, Spain; 8 Bipolar and Depressive Disorders Unit, Institute of Neuroscience, Hospital Clinic, University of Barcelona, Barcelona, Spain; 9 Department of Medicine, University of Barcelona, Barcelona, Spain; 10 Department of Medicine and Psychiatry, Universidad de Zaragoza, Zaragoza, Spain; 11 Instituto de Investigación Sanitaria Aragón (IIS Aragón), Zaragoza, Spain; 12 Hospital Universitario de Alava, Vitoria-Gasteiz, Spain; 13 Instituto de Investigación Sanitaria Bioaraba, Vitoria-Gasteiz, Spain; 14 University of the Basque Country, Vizcaya, Spain; 15 Department of Child and Adolescent Psychiatry, Institute of Psychiatry and Mental Health, Hospital General Universitario Gregorio Marañón, Instituto de Investigación Sanitaria Gregorio Marañón (IiSGM), Madrid, Spain; 16 Psychiatry Department, Institut d'Investigació Biomèdica-SantPau (IIB-SANTPAU), Hospital de la Santa Creu i Sant Pau, Universitat Autònoma de Barcelona (UAB), Barcelona, Spain; 17 Department of Child and Adolescent Psychiatry and Psychology, Clínic Institute of Neurosciences, University of Barcelona, Barcelona, Spain; 18 Institute of Neuropsychiatry and Addiction, Parc de Salut Mar, Barcelona, Spain; 19 Hospital del Mar Medical Research Institute, Barcelona, Spain

**Keywords:** Cognition, early stages, first-episode psychosis, genetics, polygenic risk score, schizophrenia

## Abstract

**Background:**

Clinical intervention in early stages of psychotic disorders is crucial for the prevention of severe symptomatology trajectories and poor outcomes. Genetic variability is studied as a promising modulator of prognosis, thus novel approaches considering the polygenic nature of these complex phenotypes are required to unravel the mechanisms underlying the early progression of the disorder.

**Methods:**

The sample comprised of 233 first-episode psychosis (FEP) subjects with clinical and cognitive data assessed periodically for a 2-year period and 150 matched controls. Polygenic risk scores (PRSs) for schizophrenia, bipolar disorder, depression, education attainment and cognitive performance were used to assess the genetic risk of FEP and to characterize their association with premorbid, baseline and progression of clinical and cognitive status.

**Results:**

Schizophrenia, bipolar disorder and cognitive performance PRSs were associated with an increased risk of FEP [false discovery rate (FDR) ⩽ 0.027]. In FEP patients, increased cognitive PRSs were found for FEP patients with more cognitive reserve (FDR ⩽ 0.037). PRSs reflecting a genetic liability for improved cognition were associated with a better course of symptoms, functionality and working memory (FDR ⩽ 0.039). Moreover, the PRS of depression was associated with a worse trajectory of the executive function and the general cognitive status (FDR ⩽ 0.001).

**Conclusions:**

Our study provides novel evidence of the polygenic bases of psychosis and its clinical manifestation in its first stage. The consistent effect of cognitive PRSs on the early clinical progression suggests that the mechanisms underlying the psychotic episode and its severity could be partially independent.

## Introduction

Schizophrenia is one of the most incapacitating psychiatric conditions worldwide (Vos et al., 2015). The usual course of the disorder is marked by psychotic episodes with positive (delusions, hallucinations) and negative symptoms (apathy, social withdrawal, avolition) as well as cognitive impairment, which results in functional disability for the individual (Millan et al., 2016). It has been well-demonstrated that interventions at early stages of the illness – that is, at the onset of first-episode psychosis (FEP) – can improve subsequent outcomes (Albert & Weibell, 2019). Thus, individuals with an FEP constitute a key group for studying the risk factors linked to the development of schizophrenia and other related disorders and its progression in terms of clinical outcome in later stages (Bernardo et al., 2019).

The accomplishment of symptomatic and functional remission is one of the major objectives in FEP interventions (Andreasen et al., 2005). Although the majority of FEP patients may show an improvement in their symptomatology after antipsychotic (AP) treatment, many continue to have long-term impairments in functioning (Amoretti et al., 2021b; Austin et al., 2013; Robinson, Woerner, McMeniman, Mendelowitz, & Bilder, 2004). Outcomes in FEP can vary on a continuum from complete remission and full recovery to more severe disease progress or worse long-term course of illness (Fusar-Poli, McGorry, & Kane, 2017). A potential reason for this variability is the intrinsic diagnostic instability of patients at FEP (Schwartz, 2000). Cognitive impairment can be found to be pre-existent to the first clinical manifestation. It has been reported that cognitive performance can depend on different factors, such as treatment with second-generation APs *v.* first-generation (Harvey, Rabinowitz, Eerdekens, & Davidson, 2005), APs dose (Ballesteros et al., 2018), the potential effects of AP medications due to excessive dopaminergic blockades (Sakurai et al., 2013) and their associated anticholinergic burden properties (Ballesteros et al., 2018), the symptomatology amelioration (Faber, Smid, van Gool, Wiersma, & van den Bosch, 2012) and/or depending on the stage of the illness (Ballesteros et al., 2018). Cognitive alterations may also persist even during remission periods (Bowie & Harvey, 2006; Chang et al., 2017; Cuesta et al., 2015) and tends to be linked to more severe negative symptomatology and functioning (Milev, Ho, Arndt, & Andreasen, 2005; Puig et al., 2017). Moreover, the cognitive reserve (CR) has become a subject of study in mental disorders, as a resilience factor based on the ability of the brain to cope with psychopathology and offset the harmful effects of the disorder (Stern, 2014). In severe mental illnesses such as schizophrenia, CR has proved to predict clinical, cognitive and functional outcomes (Amoretti et al., 2018). In addition, higher CR has also been considered a protective factor in psychiatric populations (Grande et al., 2017), and has been suggested that in schizophrenia samples, it delays the clinical diagnosis threshold and severity of symptoms (Herrero et al., 2020). Therefore, the early identification of clinical, sociodemographic and biological features may be important to identify subsets of patients with similar characteristics, facilitating personalized treatment approaches (Compton, Kelley, & Ionescu, 2014).

The genetic burden for schizophrenia has been associated with related endophenotypes – i.e. measurable and heritable components linked to the external manifestation of the disorder – in healthy relatives (Greenwood, Shutes-David, & Tsuang, 2019; Seidman et al., 2015), thus evidencing common pathophysiological mechanisms. Approaches using genetic constructs such as the polygenic risk scores (PRSs) allow us to study mental disorders and overcome some limitations of candidate-gene strategies (Assary, Vincent, Keers, & Pluess, 2018; Collins, Kim, Sklar, O'Donovan, & Sullivan, 2012). Previous studies have linked the schizophrenia and bipolar disorder PRSs with symptom severity, comorbid conditions and cognitive functioning (Mistry, Harrison, Smith, Escott-Price, & Zammit, 2018a, 2018b), further evidencing the critical role of a common genetic background between mental disorders and their clinical manifestation.

The aim of this study was to analyze the association of psychopathological and cognitive PRSs in the early progression of the clinical manifestation after an FEP. We hypothesized that PRSs reflecting a greater liability for mental disorders would be associated with psychosis onset and a slower recovery of symptoms and psychosocial functionality after the FEP. Additionally, PRSs reflecting cognitive abilities would be linked to an improved cognitive status and progression after the FEP.

## Methods

This study is part of the multicentric project ‘Phenotype–genotype interaction: application of a predictive model in first psychotic episodes’ (PEPs project). A complete description of the PEPs protocol has been published previously (Bernardo et al., 2013). This longitudinal 2-year prospective follow-up study presents clinical parameters from various assessments/visits: baseline, 2-month, 6-month, 1-year and 2-year follow-up.

### Sample

During the recruitment period (2009–2012), 335 subjects who presented an FEP and 253 healthy control subjects were included in the PEPs project. Patients included in the main project met the following inclusion criteria: aged between 7 and 35 years at recruitment; presence of psychotic symptoms of less than 12 months' duration; the ability to speak Spanish correctly and providing written informed consent. The exclusion criteria were: mental retardation according to DSM-IV-TR criteria (American Psychiatric Association, 1994); history of head trauma with loss of consciousness and presence of an organic disease with mental repercussions. Healthy controls were matched with patients according to their age (±10% of flexibility), sex and the parental socio-economic status (SES) (±1 level), determined using the Hollingshead's Two-Factor Index of Social Position, which has five potential levels: high, medium-high, medium, medium-low and low (Hollingshead & Redlich, 2007). Controls also had to be fluent in Spanish and give written informed consent. The exclusion criteria for controls were the same as for the patients, plus the presence of a present or past psychotic disorder or major depression and having a first-degree relative with psychotic disorder history.

For the present study, we identified those subjects from the PEPs cohort who provided blood samples for genetic analysis, passed the genetic quality control (see below), aged ⩾16 years old and had European ancestry. Thus, the final sample comprised of 233 FEP subjects (Table 1) and 145 healthy controls [97 males (66.9%), mean age = 24.5 years (s.d. = 5.4)]. First assessments of clinical and cognitive data were available for a range of 160–232 and complete follow-up data for a range of 89–182 FEP patients. This study was conducted in accordance with the ethical principles of the Declaration of Helsinki and Good Clinical Practice and the Hospital Clinic Ethics and Research Board. Informed consent was obtained from all participants or from parents or legal guardians of under-age subjects.

**Table 1.** Main sociodemographic, pharmacological and clinical features of the FEP sample

Feature		Mean (s.d.) or *n* (%)
Sex	Male	162 (69.5%)
	Female	71 (30.5%)
Age at FEP		24.6 (5.7)
Psychosis type	Non-affective	196 (84.1%)
	Affective	37 (15.9%)
Main AP medication at basal point	Olanzapine	78 (34.9%)
	Risperidone	66 (29.6%)
	Aripiprazole	30 (13.5%)
	Paliperidone	18 (8.1%)
	Quetiapine	13 (5.8%)
	Amisulpride	8 (3.6%)
	Clozapine	5 (2.2%)
	Haloperidol	3 (1.3%)
	Ziprasidone	1 (0.4%)
	Zuclopenthixol	1 (0.4%)
Other medication at basal point	Anxiolytic	99 (43.6%)
	Antidepressant	29 (12.8%)
	Antiepileptic	21 (9.3%)
	Lithium	15 (6.6%)
AP CEDD (12 months)		133.9 (140.8)
AP CEDD (24 months)		89.7 (95.9)
PAS		44.8 (23.9)
CR		76.7 (12.1)

AP, antipsychotic; PAS, Premorbid Adjustment Scale; CR, cognitive reserve; CEDD, chlorpromazine equivalent daily doses.

### Assessments

#### Sociodemographic, clinical and pharmacological assessments

The complete assessment of the PEPs project is reported by Bernardo et al. (2013). Within the PEPs project, a complete psychopathological assessment was carried out during the 2 years of follow-up. For the present study, due to the potential loss of sample at 2 years, we focused on symptomatology and functional data for a period of 1 year.

*General sociodemographic data and clinical assessment:* Sex, age and age at the onset of the illness were collected along with the duration of the untreated psychosis and the parental SES (Hollingshead & Redlich, 2007). The diagnosis was confirmed using the Structured Clinical Interview for Diagnostic and Statistical Manual of Mental Disorders (DSM) (SCID-I and -II) (First, Gibbon, Spitzer, Williams, & Benjamin, 1997; González-Pinto et al., 2008) according to DSM-IV criteria. The psychopathological assessment was carried out with the Spanish versions of the different scales. Symptomatology was assessed with the Positive and Negative Syndrome Scale (PANSS) (Kay, Fiszbein, & Opler, 1987; Peralta & Cuesta, 1994). Higher scores on this scale indicate greater severity. Regarding the psychosocial functioning assessment, the overall functional outcome was assessed by means of the Functioning Assessment Short Test (FAST) (Amoretti et al., 2021a; Rosa et al., 2007). The FAST scale comprises six specific areas of functioning: autonomy, occupational functioning, cognitive functioning, financial issues, interpersonal relationships and leisure time. Higher scores indicate worse functioning. The Premorbid Adjustment Scale (PAS) (Cannon-Spoor, Potkin, & Jed Wyatt, 1982) was applied retrospectively to assess premorbid adjustment. The PAS was completed based on information from patients and parents and/or close relatives. Higher scores indicate worse premorbid adjustment.

*Pharmacological assessment:* Pharmacological treatment was also collected at each visit. Chlorpromazine equivalents, expressed as chlorpromazine equivalent daily dose (CEDD), based on international consensus (Gardner, Murphy, O'Donnell, Centorrino, & Baldessarini, 2010) were calculated for AP medication. As this was a naturalistic study, there were no specific guidelines for treatment, so patients received pharmacological treatment based on the clinician's decision. Prior treatment with APs did not exceed 12 months at study entry (Bioque et al., 2016). For this study, the dose of AP was calculated as the mean CEDD.

#### Cognitive assessment

In the PEPs project, the cognitive assessment at baseline was performed in the second month after inclusion in order to ensure the clinical stability of patients after the FEP and was repeated at 2-year follow-up (Cuesta et al., 2015).

The neuropsychological battery measured the following cognitive domains: (1) sustained attention, assessed with different variables from the Continuous Performance Test-II (CPT-II) (Conners, Epstein, Angold, & Klaric, 2003), version 5; (2) verbal learning and memory, evaluated with the Verbal Learning Test Spain Complutense for adults (TAVEC) (Benedet, Christiansen, & Goodglass, 1998); (3) working memory, based on the Digit Span Subtest and the Letter-Number Sequencing Subtest of the Wechsler Adult Intelligence Scale (WAIS-III) (Wechsler, 1997) and (4) executive functioning, evaluated using the Wisconsin Card Sorting Test (WCST) (Heaton, 1993), corrected by age and educational level. Following our previous work, a principal component analysis (PCA) was performed between 10 neuropsychological variables from the battery tests aforementioned identifying the four cognitive domains described above (see online Supplementary Table S1) (Amoretti et al., 2020). Higher scores corresponded to better performance in all the cognitive domains except for attention. Additionally, a global cognitive score was obtained from the aforementioned cognitive domains (Amoretti et al., 2020). All the tests and measures used for domain summary scores are described elsewhere (Bernardo et al., 2013; Cabrera et al., 2016; Cuesta et al., 2015). To assess CR we used a ‘Cognitive reserve score’ conducted by Amoretti et al. in previous works and also framed in the PEPs project (Amoretti et al., 2016, 2018). To create this ‘Cognitive reserve score’, the three most commonly proposed proxy indicators of CR were used (Amoretti et al., 2016, 2018; Barnett, Salmond, Jones, & Sahakian, 2006; de la Serna et al., 2013; González-Ortega et al., 2019). These include IQ, education and participation in leisure, social and physical activities. Higher scores in this proxy correspond to better performance.

In the PEPs project, all clinical assessments were administered by expert clinicians after done an extensive training in each scale, except for those that were self-administered. Those who failed the first evaluation were reassessed. In the cognitive assessment, to evaluate the differences between raters, an interrater reliability study was also conducted among different neuropsychologists at each center. A good to excellent inter-rater reliability among psychologists was indicated by intraclass correlation coefficients >0.80 in two of the tests of the battery: the WAIS Vocabulary subtest and WCST, in which the final score may partially depend on the judgment of the psychologist administering and correcting the test. The complete method and the results found in the PEPs project have already been described in a specific work (Cuesta et al., 2015).

### Blood samples and genotyping

Blood samples were collected in K2EDTA BD Vacutainer EDTA tubes (Becton Dickinson, Franklin Lakes, New Jersey), stored at −20°C and sent to the central laboratory. DNA was extracted with the MagNA Pure LC DNA isolation kit – large volume and MagNA Pure LC 2.0 Instrument (Roche Diagnostics GmbH, Mannheim, Germany). DNA concentration was determined by absorbance (ND1000, NanoDrop, Wilmington, Delaware). A total of 2.5 μg of genomic DNA was sent for genotyping at the Spanish National Genotyping Centre (CeGen) using Axiom™ Spain Biobank Array (developed in the University of Santiago de Compostela, Spain).

### PRS calculation

Genotyping data were submitted to the Michigan Imputation Server (Das et al., 2016), following the standard pipeline for Minimac4 software and setting a European population reference from build GRCh37/hg19, reference panel HRC 1.1 2016 and Eagle v2.4 phasing.

For the PRS calculation, genome-wide association study (GWAS) summary results from multiple repositories (Psychiatric Genomics Consortium and SSGAC). The selected PRSs were: schizophrenia (PRS_SZ_; 69 369 cases and 236 642 controls) (Ripke, Walters, & O'Donovan, 2020), bipolar disorder (PRS_BD_; 41 917 cases; 371 549 controls) (Mullins et al., 2021), depression (PRS_DEP_; 246 363 cases; 561 190 controls) (Howard et al., 2019), education attainment and cognitive performance (PRS_EA_ and PRS_CP_; 1 131 881 and 257 841 individuals; respectively) (Lee et al., 2018). Higher psychopathological PRSs reflect a greater liability for the disorder and higher cognitive scores a better cognitive performance; duplicated and unknown strand GWAS summary single-nucleotide polymorphisms (SNPs) were excluded.

The aforementioned PRSs were selected for this study according to multiple criteria. The psychopathological PRSs (PRS_SZ_, PRS_BD_, PRS_DEP_) were chosen for their clinical proximity to an FEP and the shared genetic background among the disorders (Lee et al., 2019). On the other hand, while PRS_CP_ captures more specific cognitive abilities, PRS_EA_ also includes other personal and social abilities that reflect the academic success.

The quality control was performed with PLINK v1.07 (Purcell et al., 2007). Inclusion criteria for SNPs were minor allele frequency >0.1, Hardy–Weinberg equilibrium *p* > 10^−6^, marker missingness <0.01 and imputation INFO > 0.8. Pruning was done using a window/step size of 200/50 kb and *r*^2^ > 0.25. Sample quality control included individuals with heterozygosity values within three standard deviations (s.d.) from the mean, a missingness rate <0.01, matching chromosomal and database-labeled sex, relatedness π-hat < 0.125 and self-reported European ancestry. PRS's capacity to discriminate cases from controls and predictivity has been highly correlated with ancestry, since most reference GWAS participants are European (Perkins et al., 2020; Vassos et al., 2017).

PRSs were constructed using PRSice-2 v2.3.3 software (Choi & O'Reilly, 2019), with clumping parameters at 250 kb and *r*^2^ > 0.1 and using the odds ratio (OR) or beta values of SNPs in the reference GWAS data that had *p* < 0.05. This *p* value was used as the default threshold for the five PRSs to avoid the genetic noise of weakly associated SNPs in the reference GWAS and model overfitting (Choi, Mak, & O'Reilly, 2020). Further information about the constructed PRSs can be found in online Supplementary Fig. S1.

### Statistical analysis

All the analyses were performed with R (R Core Team, 2017). To avoid false-positive results, the false discovery rate (FDR) method was applied and the significance threshold was set at 0.05. A genetic PCA was performed to control population stratification (Patterson, Price, & Reich, 2006) by means of the SNPRelate package, and the first 10 components were used as covariates in the statistical analyses.

All PRSs were dichotomized into high risk PRS (above the highest 75% score quartile) and mid-to-low risk PRS (below the highest 75% score quartiles). This procedure was performed using the whole sample to better capture the effect of high genetic risk and avoid putative intermediate and low scores masking effect (Lin et al., 2018; Mas et al., 2020; Vassos et al., 2017; Wang et al., 2018).

The comparison of sociodemographic, pharmacological, clinical and cognitive variables between the whole FEP sample (*n* = 335) and the present study FEP sample (*n* = 233) as well as sex and age differences between FEP and controls were performed by means of chi-square and *t* tests.

The risk of the PRSs for an FEP was assessed by a chi-square test and the associated ORs. The association between basal PRS and different clinical outcomes – in terms of psychopathological symptoms, psychosocial functioning and cognitive status – was evaluated with generalized linear models corrected by sex, age, previous AP treatment days and the first 10 components of the genetic PCA. For those individuals with complete data at all assessment points, linear mixed-effects modeling was used for longitudinal analyses, considering the month of assessment as a random effect and the PRS as the fixed effect, corrected by sex, age, previous AP days, AP dose (1 year AP CEDD mean for symptomatology and functionality and 2 years AP CEDD mean for cognitive status) and the first 10 components of the genetic PCA. For linear mixed-effects models with a significant between-subject difference, post-hoc analyses were performed to characterize the effect of the PRSs at each assessment point. These analyses were performed by means of generalized linear models including sex, age, previous AP treatment days and the first 10 components of the genetic PCA as covariates.

## Results

### Descriptive statistics

The FEP sample of the present study (*n* = 233) was compared to the total FEP sample of the PEPs project (*n* = 335). The sample of the study was found representative, only different for the mean age (the sample study was 23.6 years and the total PEPs 24.6 years, *p* = 0.046) (online Supplementary Table S2). The main features of the FEP sample of the study at study entry and premorbid status are reported in Table 1 and the symptomatology, psychosocial functioning and cognitive measurements for the assessments during the follow-up in Table 2. The dropout rate of the FEP patients ranged from 19.8% to 38.4%.

**Table 2.** Clinical and cognitive assessments during the follow-up

	Basal		2-month		6-months		12-months		24-months		Available sample for longitudinal analyses
	*n*	Mean (s.d.)	*n*	Mean (s.d.)	*n*	Mean (s.d.)	*n*	Mean (s.d.)	*n*	Mean (s.d.)	
Symptomatology											
Positive	232	18.5 (8.2)	223	18.8 (5.3)	207	10.5 (4.3)	186	10.1 (4.6)	–	–	182
Negative	232	18.3 (7.9)	223	16.7 (6.8)	207	15.3 (6.4)	186	14.7 (6.5)	–	–	182
General	232	37.4 (12.6)	223	30.0 (10.4)	207	26.9 (8.7)	186	26.0 (9.6)	–	–	182
Total	232	74.2 (24.1)	223	58.5 (20.2)	207	52.7 (17.0)	186	50.8 (18.4)	–	–	182
Functionality											
Autonomy	226	4.3 (3.5)	213	3.54 (3.1)	200	3.25 (2.9)	177	2.86 (2.9)	–		158
Occupational functioning	226	7.9 (5.5)	213	7.1 (5.2)	200	6.1 (5.2)	177	5.6 (5.2)	–	–	158
Cognitive functioning	226	5.8 (3.9)	213	4.8 (3.7)	200	3.8 (3.3)	177	3.6 (3.4)	–		158
Financial issues	226	1.5 (1.8)	213	1.2 (1.6)	200	0.9 (1.4)	177	0.9 (1.4)	–	–	158
Interpersonal relationships	226	6.7 (4.9)	213	5.6 (4.5)	200	4.9 (4.3)	177	4.5 (4.3)	–	–	158
Leisure time	226	2.1 (1.8)	213	2.0 (1.8)	200	1.8 (1.7)	177	1.7 (1.6)	–	–	158
Total	226	28.0 (16.4)	213	24.2 (15.3)	200	20.8 (14.8)	177	19.1 (14.5)	–	–	158
Cognitive status											
Attention	–	–	166	88.6 (8.9)	–	–	–	–	104	86.0 (9.5)	93
Working memory	–	–	188	79.8 (16.0)	–	–	–	–	115	84.1 (15.7)	114
Verbal memory	–	–	181	134.0 (50.4)	–	–	–	–	112	159.0 (47.7)	107
Executive function	–	–	177	126.0 (43.7)	–	–	–	–	109	150.0 (41.6)	102
Composite score	–	–	160	294.0 (50.2)	–	–	–	–	99	330.0 (49.0)	89

### FEP risk

There were no age or sex differences in the FEP individuals and controls (*p* = 0.908, *p* = 0.637; respectively). All PRSs were used to assess their association with the risk of suffering an FEP in our cohort. There was a higher proportion of high risk PRS_SZ_ [31.8% *v.* 15.2%, FDR = 0.004, OR (95% CI) = 2.60 (1.53–4.42)] and PRS_BD_ [29.2% *v.* 17.2%, FDR = 0.028, OR (95% CI) = 1.98 (1.18–3.31)] and a lower proportion of high risk PRS_CP_ [20.2% *v.* 32.4%, FDR = 0.034, OR (95% CI) = 0.53 (0.33–0.85)] in FEP individuals (online Supplementary Table S1). Thus, high scores PRS_SZ_ and PRS_BD_ conferred an increased risk of FEP and high scores for PRS_CP_ had a protective effect.

### Baseline analysis

Symptomatology, psychosocial functionality and cognitive status were evaluated at baseline for the FEP patients. No significant effects of the PRSs were found for the baseline measurement of symptoms and functionality. As for the cognitive status, higher PRS_DEP_ was found to be associated with decreased executive function (FDR = 0.019), higher PRS_EA_ and PRS_CP_ with an increased working memory (FDR = 0.039, FDR = 0.024; respectively) and with an increased CR (FDR = 0.037, FDR = 0.001; respectively) (Table 3). Baseline association analyses of clinical status and PRSs constructed with different *p* value thresholds can be found in online Supplementary Table S4.

**Table 3.** Basal association of PRSs with clinical scales, cognitive status and premorbid adjustment

	PRS_SZ_				PRS_BD_				PRS_DEP_				PRS_EA_				PRS_CP_			
	Estimate	*t*	*R*^2^	FDR	Estimate	*t*	*R*^2^	FDR	Estimate	*t*	*R*^2^	FDR	Estimate	*t*	*R*^2^	FDR	Estimate	*t*	*R*^2^	FDR
Symptomatology																				
Positive	0.451	0.330	0.061	0.742	−1.098	−0.782	0.063	1.000	0.919	0.657	0.062	0.768	1.985	1.457	0.070	0.147	2.332	1.684	0.074	0.188
Negative	−0.512	−0.384	0.051	0.832	0.409	0.298	0.051	0.967	−1.160	−0.852	0.053	1.000	1.625	1.222	0.059	0.357	1.557	1.150	0.058	0.222
General	0.647	0.317	0.061	1.000	−0.541	−0.256	0.060	1.000	−0.375	−0.179	0.059	0.945	3.817	1.869	0.072	0.206	3.112	1.485	0.065	0.267
Total	0.786	0.195	0.050	1.000	−1.995	−0.482	0.050	1.000	−0.568	−0.138	0.049	0.975	6.643	1.659	0.065	0.152	6.009	1.473	0.061	0.124
Functionality																				
Autonomy	0.125	0.208	0.035	0.835	1.154	1.909	0.054	0.173	−0.477	−0.774	0.038	0.660	0.714	1.202	0.043	0.462	0.499	0.822	0.039	0.412
Occupational functioning	0.131	0.144	0.078	0.886	0.695	0.750	0.081	1.000	−0.667	−0.713	0.081	0.715	−0.791	−0.875	0.082	0.765	−0.138	−0.150	0.078	0.881
Cognitive functioning	1.045	1.519	0.033	0.392	0.545	0.778	0.024	0.656	0.029	0.041	0.021	0.967	0.842	1.234	0.029	0.438	0.828	1.191	0.028	0.235
Financial issues	0.425	1.670	0.053	0.353	0.178	0.691	0.050	0.370	−0.050	−0.186	0.055	0.541	0.026	0.103	0.046	0.844	0.375	1.443	0.049	0.881
Interpersonal relationships	1.082	1.267	0.032	0.625	0.904	1.058	0.040	0.546	−0.460	−0.527	0.035	0.628	0.264	0.316	0.033	1.000	0.666	0.776	0.033	0.554
Leisure time	−0.543	−1.791	0.073	0.248	0.328	1.054	0.065	0.518	−0.470	−1.507	0.079	0.216	0.644	2.128	0.075	0.246	0.250	0.817	0.067	0.357
Total	1.675	0.587	0.046	0.558	4.062	1.411	0.054	0.480	−2.838	−0.974	0.049	0.497	1.837	0.650	0.046	0.516	2.244	0.780	0.047	0.872
PAS	−2.022	−0.525	0.139	0.544	3.795	0.953	0.142	0.440	−6.333	−1.565	0.148	0.372	7.278	1.868	0.156	0.081	7.084	1.791	0.149	0.105
Cognitive status																				
Attention	−2.082	−1.343	0.182	0.629	0.810	0.524	0.173	0.698	−0.523	−0.307	0.175	0.814	2.040	1.310	0.174	0.627	3.469	2.227	0.195	0.115
Working memory	2.768	1.022	0.196	0.925	−2.444	−0.880	0.194	0.571	2.022	0.691	0.193	0.490	−5.680	−2.087	0.213	0.039	−6.936	−2.546	0.223	0.024
Verbal memory	8.978	0.984	0.115	0.490	−5.716	−0.620	0.111	0.536	16.350	1.691	0.126	0.279	−8.302	−0.896	0.114	0.372	−9.842	−1.063	0.116	0.579
Executive function	2.841	0.348	0.108	0.893	2.326	0.280	0.108	1.000	25.468	3.064	0.153	0.019	−1.599	−0.201	0.108	0.967	−4.188	−0.510	0.111	0.978
Composite score	0.409	0.469	0.175	0.645	1.156	1.313	0.179	0.565	−0.654	−0.726	0.206	0.075	0.610	0.703	0.176	0.554	0.590	0.668	0.180	0.634
CR	1.641	0.822	0.186	0.425	−2.103	−1.022	0.192	0.380	1.611	0.752	0.183	0.440	−4.654	−2.329	0.203	0.037	−6.652	−3.342	0.247	0.001

SZ, schizophrenia; BD, bipolar disorder; DEP, depression; EA, education attainment; CP, cognitive performance; PAS, Premorbid Adjustment Scale; CR, cognitive reserve.

Significant results are marked in bold.

Corrected by sex, age, previous AP treatment days and first 10 components of genetic PCA.

### Longitudinal analysis

**Fig. 1.**
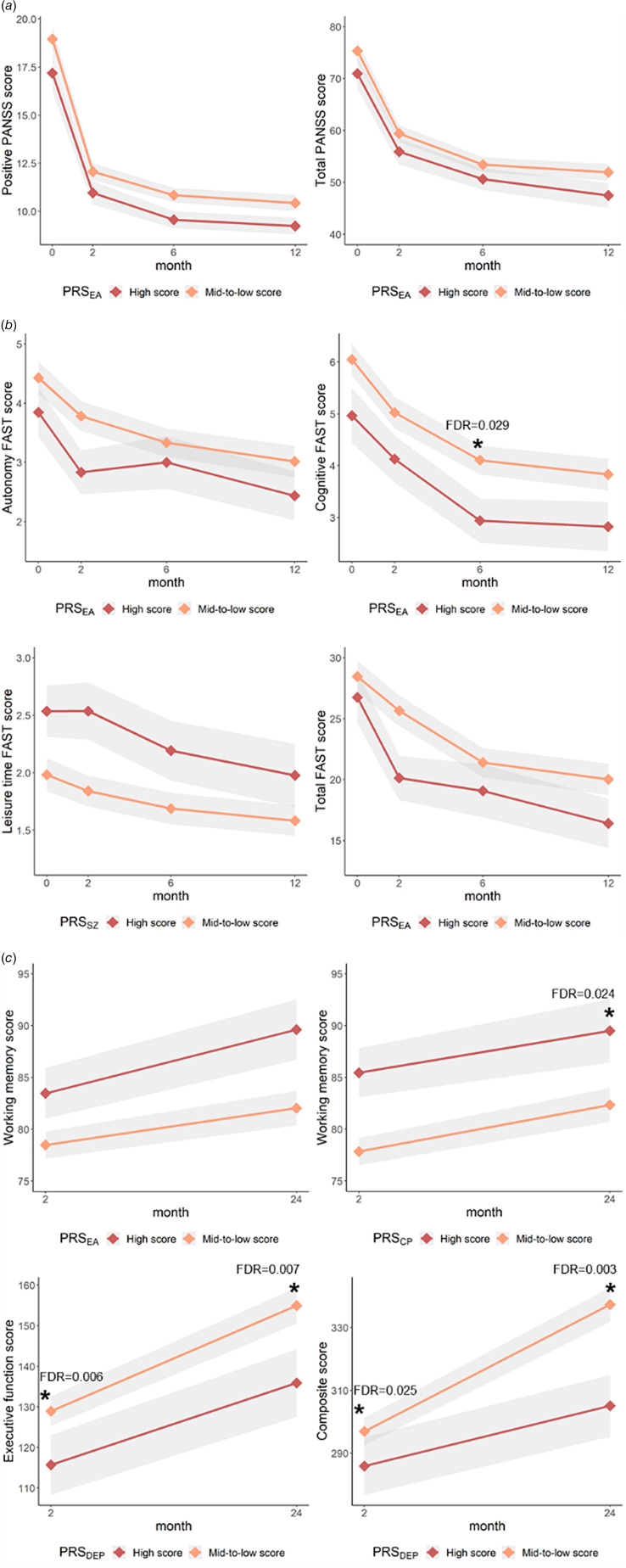
Summary of the progression of clinical measures during follow-up. The plots show the mean of each clinical measurement and standard error range for each month of assessment. (a) Symptomatology progression, (b) psychosocial functionality progression and (c) cognitive progression. Significant post-hoc analyses are marked with an asterisk. DEP, Depression; EA, education attainment; CP, cognitive performance.

Follow-up clinical data were used for the longitudinal analyses. Increased PRS_EA_ was associated with trajectories reflecting the manifestation of less positive and total PANSS symptoms (FDR = 0.019, FDR = 0.026; respectively), but no post-hoc differences were found, thus showing no significant effect of the PRS_EA_ on symptom severity at any discreet assessment point. Additionally, a trend of an association of PRS_CP_ and positive symptom progression was found (FDR = 0.051) (Fig. 1a; Table 4).

**Table 4.** Longitudinal association of PRSs with clinical scales and cognitive status

	PRS_SZ_				PRS_BD_				PRS_DEP_				PRS_EA_				PRS_CP_			
	Estimate	*t*	*R*^2^	FDR	Estimate	*t*	*R*^2^	FDR	Estimate	*t*	*R*^2^	FDR	Estimate	*t*	*R*^2^	FDR	Estimate	*t*	*R*^2^	FDR
Symptomatology																				
Positive	−0.304	−0.360	0.282	1.000	−0.958	−1.100	0.284	0.820	−0.195	−0.217	0.281	0.829	2.231	2.636	0.299	0.019	1.723	1.970	0.291	0.051
Negative	0.200	0.188	0.119	0.851	0.718	0.652	0.120	0.773	−1.028	−0.908	0.121	1.000	1.951	1.809	0.130	0.145	1.738	1.573	0.127	0.118
General	0.253	0.158	0.216	0.874	−1.854	−1.128	0.220	0.784	−0.637	−0.375	0.216	1.000	3.512	2.184	0.231	0.061	2.498	1.506	0.223	0.134
Total	0.156	0.051	0.242	0.959	−2.090	−0.661	0.243	1.000	−1.848	−0.567	0.243	0.857	7.703	2.509	0.261	0.026	5.973	1.886	0.253	0.061
Functionality																				
Autonomy	−0.269	−0.553	0.081	0.871	0.481	0.960	0.083	1.000	−0.220	−0.426	0.081	0.671	1.382	2.852	0.110	0.010	0.854	1.700	0.091	0.091
Occupational functioning	−0.219	−0.277	0.100	0.782	0.557	0.681	0.101	1.000	0.386	0.460	0.100	0.969	0.044	0.054	0.099	0.957	0.190	0.230	0.099	1.000
Cognitive functioning	0.115	0.225	0.103	1.000	−0.024	−0.046	0.103	0.964	0.139	0.257	0.103	1.000	1.526	3.017	0.130	0.006	0.895	1.694	0.112	0.092
Financial issues	−0.031	−0.127	0.088	0.899	0.217	0.857	0.091	1.000	−0.182	−0.699	0.090	0.728	0.517	2.087	0.103	0.077	0.343	1.347	0.094	0.180
Interpersonal relationships	−0.769	−1.091	0.090	0.831	−0.130	−0.178	0.086	0.859	−0.663	−0.884	0.088	0.568	1.585	2.227	0.104	0.055	0.449	0.609	0.087	0.544
Leisure time	−0.636	−2.621	0.099	0.029	0.086	0.334	0.077	1.000	0.002	0.008	0.077	0.994	0.468	1.859	0.088	0.130	0.323	1.250	0.082	0.213
Total	−1.763	−0.765	0.124	1.000	1.199	0.501	0.122	0.925	−0.527	−0.215	0.122	0.830	5.476	2.359	0.142	0.039	3.073	1.281	0.127	0.202
Cognitive status																				
Attention	−2.551	−1.622	0.258	0.323	1.790	1.109	0.254	0.270	−2.185	−1.200	0.255	0.349	2.966	1.863	0.264	0.130	1.725	1.052	0.251	0.295
Working memory	3.380	1.218	0.181	0.677	−3.012	−1.050	0.180	0.444	1.408	0.454	0.175	0.444	−9.931	−3.693	0.242	0.001	−6.216	−2.203	0.197	0.030
Verbal memory	11.851	1.283	0.139	0.303	−8.943	−0.936	0.137	0.351	20.324	2.019	0.155	0.137	−13.414	−1.435	0.136	0.308	−9.492	−0.997	0.162	0.321
Executive function	2.277	0.280	0.162	0.780	−3.274	−0.397	0.163	1.000	35.197	4.295	0.247	1.08 × 10^−4^	10.113	1.266	0.169	0.416	0.053	0.006	0.162	0.995
Composite score	7.960	0.806	0.232	0.422	−13.191	−1.325	0.242	0.282	40.301	3.799	0.302	0.001	−7.818	−0.790	0.233	0.863	−7.294	−0.729	0.233	0.468

SZ, schizophrenia; BD, bipolar disorder; DEP, depression; EA, education attainment; CP, cognitive performance.

Significant results are marked in bold.

Corrected by sex, age, previous AP days and AP dose (1 year AP CEDD mean for symptomatology and functionality and 2 years AP CEDD mean for cognitive status) and first 10 components of genetic PCA.

Regarding the psychosocial functionality progression, higher PRS_EA_ was associated with trajectories reflecting an increased autonomy, cognitive functioning and a lower total score (FDR = 0.010, FDR = 0.006, FDR = 0.039; respectively). A trend of an association of PRS_EA_ and the financial issues was found (FDR = 0.055). Higher PRS_SZ_ was associated with a worse progression of the leisure time domain (FDR = 0.029). Post-hoc differences were found for PRS_EA_ and cognitive functioning at month 6 (FDR = 0.029) (Fig. 1b; Table 4).

Cognitive measurements were also used for longitudinal assessment. Higher PRS_EA_ and PRS_CP_ were associated with trajectories reflecting an increased working memory (FDR = 0.001, FDR = 0.030; respectively) and higher PRS_DEP_ with a decrement of the executive function and the composite score (FDR = 1.08 × 10^−4^, FDR = 0.001; respectively). Post-hoc differences were found for PRS_CP_ and working memory at month 24 (FDR = 0.024) and for PRS_DEP_ and the executive function at baseline and month 24 (FDR = 0.006, FDR = 0.007; respectively) and for the composite score at baseline and month 24 (FDR = 0.025, FDR = 0.003; respectively) (Fig. 1c; Table 4). Longitudinal association analyses of clinical status and PRSs constructed with different *p* value thresholds can be found in online Supplementary Table S5.

## Discussion

### Main findings

Early intervention at the initial manifestation of severe mental disorders is critical to prevent poor outcomes, and therefore the characterization of factors associated with the prognosis such as genetics are key to understand the underlying mechanisms. The present study aimed to investigate the role of the genetic burden for psychopathological disorders and cognitive features in the clinical progression after an FEP. The PRS reflecting the cognitive performance was associated with the CR. Moreover, educational attainment, cognitive performance and depression PRSs were associated with the course of symptoms, psychosocial functioning and the cognitive status after the psychosis onset. It is noteworthy that increased PRSs for schizophrenia and bipolar disorder conferred an increased risk of suffering an FEP but did not influence symptomatologic or cognitive parameters, providing evidence that early symptom improvement might be partially independent from the psychopathological mechanisms that determine the onset of psychosis.

### Schizophrenia PRS

PRSs calculated with schizophrenia GWAS have been widely associated with risk of psychopathology development in chronic and FEP samples (Perkins et al., 2020; Santoro et al., 2018; Sørensen et al., 2018; Toulopoulou et al., 2019; Vassos et al., 2017; Wang et al., 2018; Zheutlin et al., 2019). To the best of our knowledge, this is the first study to replicate these previous findings using PRSs constructed with the third and largest wave of the Psychiatric Genomics Consortium (Ripke et al., 2020). Previous findings report inconsistent associations with clinical features such as symptom severity, neurocognitive performance and treatment resistance (Chen et al., 2018; Jonas et al., 2019; Ohi et al., 2018; Perkins et al., 2020; Richards et al., 2020; Santoro et al., 2018; Shafee et al., 2018; Sørensen et al., 2018; Werner et al., 2020; Wimberley et al., 2017; Zhang et al., 2019), possibly due to the heterogeneity of samples in terms of schizophrenia progression and AP treatment consequences. Considering the lack of association of PRS_SZ_ with clinical or cognitive features in our FEP sample (only with the recovery of leisure time functionality domain) and otherwise positive associations in the literature, we cannot rule out the possibility that this PRS could have a role for some specific clinical manifestations – e.g. a greater number of psychotic episodes, an earlier age at onset or worse response to treatment – that lead to a debilitating and chronic course, recognizable in latter stages several years after the onset of the disorder.

### Bipolar disorder PRS

The effect of bipolar disorder PRSs in schizophrenia has been described in multiple studies (Mistry et al., 2018a), but no previous information about its role on FEP risk can be found in the literature. Here, we report for the first time the risk of PRS_BD_ to develop an FEP. Similarly to the PRS_SZ_, we could not find any effect of PRS_BD_ on the clinical and cognitive status, in accordance with the study of Richards et al. (2020). On the other hand, no association of PRS_DEP_ with FEP risk could be found. Yet, worse scores of this PRS were linked to impaired cognitive status after an FEP. Our findings could be capturing the defective cognitive functionality associated with the impaired dysfunctional goal-directed decision-making processes and reward maximization found in mood disorders (Saperia et al., 2019).

### Cognitive PRSs

Impaired cognitive functions of schizophrenia patients can be found before illness onset and therefore they are not entirely a consequence of the psychotic (Ayesa-Arriola et al., 2021). This places abnormal neurodevelopment as a core component in the onset of schizophrenia (Kobayashi et al., 2014) while also suggesting a genetic etiology (Dickinson et al., 2020). In order to delve into the genetic foundations of the clinical and cognitive manifestation of our FEP sample, two scores reflecting the cognitive performance of the general population were calculated. While PRS_CP_ specifically captures the genetic basis for neurocognitive capacities, PRS_EA_ – based on the years of schooling and comprising >1.1 million individuals – also relates to social, economic and health outcomes (Lee et al., 2018). For the first time we are able to describe a protective effect of the genetics underlying cognitive features in the early progression of clinical manifestation after an FEP. At study entry, the effect of cognitive PRSs could only be detected on the cognitive status. Nonetheless, the role of PRS_EA_ on the evolution of symptom severity and functionality suggests that the protective factor of the cognitive PRS may have a more relevant role in symptom and functionality regain. Regarding the cognitive progression, the protective effect of cognitive PRSs on the working memory domain agrees with the work of Richards and colleagues, in which a very strong link between the cognitive PRSs and the general intelligence factor is reported (Richards et al., 2020).

### Cognitive reserve

The premorbid cognitive status (measured as CR) has been proposed as a mediator between the clinical manifestation and the final psychosocial functioning, possibly acting as a coping mechanism for the long-term effects on patients (Amoretti et al., 2020). CR has been consistently identified as baseline and 2-year mediator of symptomatology, functionality and cognition in previous studies of the PEPs project (Amoretti et al., 2016, 2018, 2020; González-Ortega et al., 2019). In the present work, the PRS_CP_ was associated with a better cognitive progression, higher FEP risk as well as with an increased CR. Moreover, it has been demonstrated in our previous studies that having a high CR and better premorbid adjustment may confer a better prognosis (Amoretti et al., 2021b). If the role of CR as mediator of symptomatology, functionality and cognition is confirmed and the association of cognitive PRSs with CR is replicated in independent cohorts, it could be considered that individuals with increased a genetic basis for a better cognition would be more resilient to the distressful effects of the psychotic episode and have a better prognosis.

### Limitations and strengths

Some limitations of the present work should be taken into consideration. First, sample size is moderately limited in the longitudinal follow-up due to patient drop-out and therefore the statistical analysis might be underpowered to detect small effects. In addition, due to constraints associated with the PANSS (Blanchard, Kring, Horan, & Gur, 2011), another limitation of the study has been the absence of a specific scale to assess negative symptomatology, such as the Brief Negative Symptom Scale (BNSS) (Kirkpatrick et al., 2011; Mané et al., 2014) or a specific tool to assess the CR, as at the time that the PEPs project was developed (2009–2012) there was no validated instrument to measure the CR as the Cognitive Reserve Assessment Scale in Health (CRASH) (Amoretti et al., 2019) and the BNSS was under development. However, this study comprises one of the largest and best characterized FEP samples in the literature, with a naturalistic design and thus representative of the psychiatric population without the confounding effect of prolonged AP treatment, medical comorbidities or chronicity. The subsample used for the present study is comparable with the total PEPs sample, with the exception of a small difference of mean age (most probably due to age restriction criteria). The PRSs have been calculated with the largest GWAS from international consortiums and thus the comprised genetic variants have a great capacity to capture the genetic susceptibility of the phenotypes. Strict quality control of genetic data and multiple test significance thresholding have been implemented to prevent methodological artifacts and statistical errors in the results.

## Conclusions

Novel genetic approaches considering the polygenic etiology of psychotic disorders are crucial to disentangle the molecular basis of the pathophysiological mechanisms underlying the onset and progression of schizophrenia. Cognitive rather than psychopathological polygenic scores were found widely associated to premorbid cognitive status and symptom recovery, suggesting that the underlying mechanisms mediating the emergence of the psychotic episode and its severity could be partially independent. Further research on this topic is essential to unravel the etiopathogenic processes of schizophrenia to ultimately prompt early intervention protocols for high-risk individuals and provide personalized attention – both pharmacological and psychological – to prevent severe forms of the disorder.

## Supplementary material

For supplementary material accompanying this paper visit https://doi.org/10.1017/S0033291722001544. click here to view supplementary material
